# Relationship Between Paraspinal Muscle Degeneration and Functional Outcomes Following Anterior Cervical Spine Surgery for Degenerative Disk Disease: A Systematic Review

**DOI:** 10.3390/jcm14238453

**Published:** 2025-11-28

**Authors:** Jan Chrzanowski, Tomasz A. Dziedzic, Przemyslaw Kunert

**Affiliations:** Department of Neurosurgery, Medical University of Warsaw, Zwirki i Wigury 61, 02-091 Warsaw, Poland

**Keywords:** paraspinal muscles, cervical spine surgery, degenerative disk disease

## Abstract

**Background/Objectives:** Paraspinal muscles are important for maintaining cervical spine function and stability; however, the degeneration of these muscles is common in patients with degenerative disk disease. Such muscular changes may affect recovery trajectories and long-term functional outcomes after cervical spine surgery. This systematic review explores the existing literature on the relationship between the degree of paraspinal muscle degradation and functional outcomes following anterior cervical spine surgery in patients with cervical degenerative disk disease. **Methods:** A systematic review of the MEDLINE/Pubmed, Web of Science, and Embase databases was conducted according to the PRISMA guidelines up to June 2025. The inclusion criteria were patients who underwent surgery for cervical degenerative disk disease and assessments of the paraspinal muscles with magnetic resonance imaging. The methodological quality of the included studies was assessed using the Modified Newcastle–Ottawa Scale. **Results:** Following deduplication, a total of 3643 articles were screened, of which 6 met the inclusion criteria and were included in the review. Across these studies, a total of 515 patients were followed for at least one year. Two studies reported a negative association between paraspinal muscle degeneration and functional outcomes, three reported no association, and one reported a positive association. **Conclusions:** The available evidence on this topic is inconclusive. These mixed results highlight the need for further well-designed, adequately powered studies to clarify the relationship between paraspinal muscle degeneration and functional outcomes.

## 1. Introduction

Cervical degenerative disk disease (CDDD) is a common spinal condition closely associated with aging. Radiological evidence of cervical spondylosis has been observed in approximately 60% of individuals older than 40 years, highlighting the widespread prevalence of this degenerative process [1]. The initial management of CDDD typically involves nonoperative interventions, such as physical therapy, pharmacological treatment, and lifestyle modifications. However, surgical treatment may be necessary in cases where conservative approaches fail or when progressive neurological deficits develop.

With aging populations in developed countries, the incidence of cervical spine surgeries is expected to increase in the coming decades [2]. Despite its generally favorable outcomes, studies have indicated that up to 20% [3] of patients report dissatisfaction following anterior cervical discectomy and fusion (ACDF), making the optimization of postoperative functional outcomes a key concern. Multiple studies have attempted to identify potential factors that influence functional outcomes following anterior cervical spine procedures [4,5,6]. One such factor that has garnered increasing attention is the condition of the paraspinal muscles.

The paraspinal muscles can be evaluated using preoperative MRI, enabling the assessment of muscle atrophy and fat infiltration [7]. In the cervical spine, structural changes in the deep paraspinal muscles—such as increased fat infiltration—have been associated with greater degrees of spinal degeneration, sagittal imbalance, and chronic neck pain [7]. To our knowledge, only one review article from 2021 has explored the role of the paraspinal muscles in cervical spine surgery [8]. However, that study did not focus primarily on functional outcomes, and since then, several additional studies have been published, offering new insights into this topic. This systematic review aims to synthesize the current evidence on how the preoperative condition of paraspinal muscles influences recovery and long-term functional outcomes following anterior cervical spine surgery. We chose to focus on anterior cervical approaches, as a previous study has demonstrated that they tend to spare the deep extensor muscles, compared with posterior techniques [9].

## 2. Materials and Methods

As a systematic review based on data from published studies, this work did not require approval from an ethical standards committee. This review followed the Preferred Reporting Items for Systematic Reviews and Meta-Analyses (PRISMA) guidelines and was not registered in PROSPERO or any other database (Supplemental Material Table S2 PRISMA Checklist).

### 2.1. Eligibility Criteria

Only articles that met the following criteria were included: (1) included patients who underwent surgery for cervical degenerative disease using the anterior approach; (2) included patients who were older than 18 years; and (3) included patients whose paraspinal muscles were assessed using conventional MRI. We excluded studies that did not assess the paraspinal muscles preoperatively; studies on surgeries for cervical spine trauma, tumors, or infection; and studies that did not provide primary data, e.g., reviews, opinions, editorials, or studies on animals.

### 2.2. Information Sources and Search Strategy

We searched Embase, MEDLINE/Pubmed, and Web of Science for reports published in English up to 31 July 2025, using the title, abstract, or key word search terms: (cervical spine surgery) or (anterior cervical discectomy and fusion) or (anterior approach) or (ACDF) or (anterior cervical decompression) or (cervical discectomy) or (cervical disk arthroplasty) and (paraspinal muscle) or (semispinalis cervicis) or (semispinalis capitis) or (multifidus) or (fat infiltration) or (posterior cervical extensor muscle) or (fatty degeneration).

Databases were screened independently by one author through the titles and abstracts of the manuscripts; studies were subsequently selected according to the previously mentioned eligibility criteria and then fully reviewed by two authors. In cases of disagreement, a third author served as a referee. Duplicate and irrelevant studies were excluded using EndNote. The bibliographies of the selected studies were also examined to identify potentially relevant references. To assess the quality of the publications, we used the Modified Newcastle–Ottawa Scale. Studies that achieved a score of 7 to 9 points, 4 to 6 points, and 3 or fewer points were considered to have a low, moderate, and high risk of bias, respectively. We utilized the North American Spine Society’s scale to assess the articles’ level of evidence (https://www.spine.org/Documents/ResearchClinicalCare/LevelsOfEvidence.pdf) (accessed on 10 September 2025). Disagreements were discussed by the reviewers until a consensus was reached.

### 2.3. Data Items

From all the selected studies, we extracted the following data: the name of the first author, year of publication, number of patients, study design, type of surgery, patient population, results, outcome measurement, and key findings.

### 2.4. Outcomes

A descriptive approach was used to summarize the main characteristics, methodologies, and findings of the studies. Owing to significant heterogeneity among the studies, a meta-analysis could not be performed.

## 3. Results

### 3.1. Study Selection

**Figure 1:**
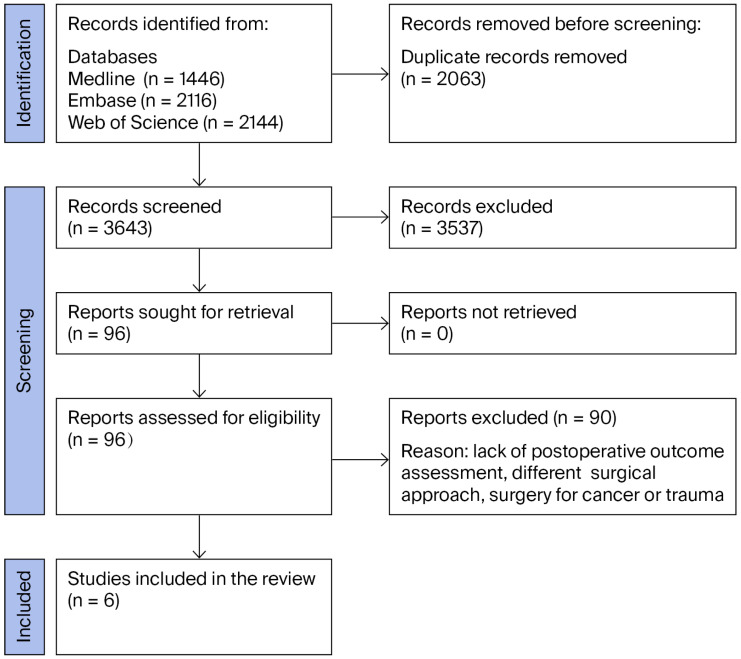
Study flow diagram.

Figure 1 presents the flowchart of the study selection according to PRISMA. We included 6 studies from the 3643 initially identified through our initial database search. Due to our eligibility criteria, we excluded works assessing muscles in trauma or oncologic patients, as well as those using posterior surgical approaches. Although this reduced the number of studies included, it ensured that our results are specific and directly applicable to the population and surgical approach of interest, thereby enhancing the validity of our conclusions.

### 3.2. Study Characteristics

Across all the studies, a total of 515 patients with at least 1 year of follow-up were included. According to the Modified Newcastle–Ottawa Scale, two studies had a moderate risk of bias, and four studies had a low risk of bias (Supplementary Materials Table S1).

All six works investigated the impact of the state of the paraspinal muscles on functional outcomes in patients treated surgically via the anterior approach for cervical spine degenerative disease. One prospective study and five retrospective studies were included. In two studies, the interventions included ACDF with a plate, while in two others ACDF was performed without a plate; one work described patients after corpectomy with fusion and plating, and another described patients following hybrid surgery. The study characteristics are presented in Table 1.

### 3.3. Muscle Assessment

All the studies assessed the paraspinal muscles using T2-weighted MRI; however, they differed in their measurement methods. Three studies utilized the Goutallier scale, two calculated the ratio between the CSA of the muscles and the VBAs, and one used the functional cross-sectional area of the muscles as a percentage of the FI.

### 3.4. Functional Assessment and Outcomes

Caffard et al. [10] retrospectively evaluated the outcomes of 168 patients who underwent ACDF, with a one-year follow-up. The NDI was used as the primary measure of the functional outcome. This was the only study that utilized qualitative assessments of the fat infiltration within the paraspinal muscles. The authors reported that the severity of FI in the posteromedial muscles at the C7 level was significantly negatively correlated with the improvement in the NDI at 4–6 months postoperatively (*p* = 0.03).

The study by Pinter et al. [11] retrospectively assessed the association between the condition of the cervical paraspinal muscles and functional outcomes following ACDF, with a minimum follow-up of one year. This investigation included 69 patients, with a mean age of 53.1 years, of whom 50.7% were male. Patients were grouped on the basis of the Goutallier grade: there were 29 patients in Group 1 (Goutallier 0–1), 29 in Group 2 (Goutallier 1.5–2), and 11 in Group 3 (Goutallier 2.5–4.0). No significant differences were observed between the groups in terms of sex, age, BMI, or comorbidities, such as hypertension, diabetes, and hyperlipidemia. Compared to patients with lower grades of paraspinal muscle degeneration, patients with higher Goutallier grades demonstrated a greater improvement in the NDI (*p* = 0.02). However, no statistically significant differences were observed in other patient-reported outcome measures, including the scores on the RAND and EQ-5D questionnaires (*p* > 0.05 and *p* = 0.07, respectively).

Wang et al. [12] retrospectively investigated the impact of the condition of the paraspinal muscles—specifically the Goutallier grade of fatty infiltration—on outcomes following ACDF. This study included 101 patients, with a mean age of 50.89 years (range: 21–78) and a mean follow-up duration of 18.52 months (range: 12–75 months). Patients were divided into three groups, similarly to those in the previous study: Group A (Goutallier grade 0–1, *n* = 33), Group B (Goutallier grade 1.5–2.0, *n* = 44), and Group C (Goutallier grade 2.5–4.0, *n* = 24). The groups differed significantly in terms of the mean age (*p* < 0.007). This study revealed no significant correlation between paraspinal muscle fatty infiltration and postoperative outcomes within one year. There were no statistically significant differences in the NDI (*p* = 0.941), VAS scores (*p* = 0.131), or JOA scores (*p* = 0.941) among the groups.

Thakar et al. [13] conducted a prospective study involving 45 patients with single-level, symptomatic cervical degenerative disk disease who presented with radiculomyelopathy or myelopathy. The mean age of the participants was 40.22 years, and 89% were male. The mean follow-up duration was 20.02 ± 8.63 months. In this study, an improvement in the Nurick grade was used as the clinical outcome measure, and paraspinal muscles were assessed by calculating the ratio between the CSA of the muscles and the VBA. The results demonstrated that a larger deep flexor CSA and a higher deep flexor/deep extensor area ratio were positively correlated with improved functional outcomes following anterior cervical discectomy (*p* = 0.01).

He et al. [14] retrospectively reviewed 110 patients (mean age: 48.8 years; range: 29–67 years; 35% male) who underwent hybrid surgery for CDDD with symptomatic radiculopathy and/or myelopathy at two contiguous levels from C3 to C7. The mean follow-up duration was 23.4 months (range: 12–93 months). Patients were grouped on the basis of the Goutallier classification of fatty infiltration: the normal group (grades 0–1, *n* = 34), the moderate group (grades 1.5–2, *n* = 38), and the severe group (grades 2.5–4, *n* = 38). The groups were matched, and no significant differences were found in regard to age, sex, or body mass index (*p* > 0.05). All groups showed significant clinical improvements following surgery. However, this study revealed no correlation between the degree of fatty infiltration in the paraspinal muscles and postoperative outcomes. There were no statistically significant differences in JOA scores (*p* = 0.923), the NDI (*p* = 0.327), or VAS scores (*p* = 0.977) among the groups.

Thakar et al. [15] retrospectively enrolled 67 patients with cervical spondylotic myelopathy, with a mean age of 52 ± 10.37 years, 91% of whom were male. A paraspinal muscle assessment was performed using MRI by calculating the CSA of the paraspinal muscles relative to the corresponding VBA, as well as the flexor/extensor CSA ratio. These measurements were compared to those of a sex- and age-matched control cohort with normal cervical spine MRIs. This study revealed that there was no significant correlation between the degeneration of the paraspinal muscle and improvements in the Nurick grade.

## 4. Discussion

This systematic review identified six studies that investigated the impact of the condition of the paraspinal muscles on functional outcomes following anterior cervical spine surgery for CDDD. Existing studies report inconsistent findings regarding this topic.

Two studies concluded that the degeneration of the paraspinal muscles was associated with worse functional outcomes. This may be related to the fact that the fatty infiltration (FI) of the paraspinal muscles has been linked to cervical spondylosis and disk degeneration, as measured by the Pfirrmann scale [16]. These degenerative changes can compromise the anterior column of the spine, increasing the load on the intervertebral joints and contributing to neck pain. Furthermore, paraspinal muscle degeneration may affect the cervical sagittal balance, which is clinically relevant, as some studies have shown that poor clinical outcomes following ACDF are associated with altered sagittal parameters such as the C2-7 sagittal vertical axis (SVA), T1 slope, and spino-cranial angle [17,18]. Additionally, structural alterations such as FI can contribute to spinal imbalance, as the paraspinal muscles are estimated to provide approximately 80% of the cervical spine’s stability [19]. Wong et al. [20] demonstrated that the preoperative morphology and composition of paraspinal muscles can predict the early onset of adjacent segment disease (ASD) following ACDF. These changes may contribute to increased postoperative pain and disability.

Half of the identified studies did not find an association between the condition of the paraspinal muscles and functional outcomes following anterior cervical spine surgery. Notably, these studies differed in their methods of muscle degeneration analysis, employing qualitative, semiquantitative, or quantitative assessments. This distinction is important, as previous research has shown that there is no association between the CSA of the paraspinal muscles and the degree of FI, highlighting the need for the careful selection of evaluation methods [21]. Caffard et al. [10] demonstrated that the severity of FI in the multifidus muscle at the C7 level was associated with a smaller improvement in NDI scores. This study utilized a quantitative analysis, which appears to offer greater precision than the semiquantitative, observer-dependent Goutallier scale. The Goutallier grading system categorizes muscle degeneration into four levels on the basis of the extent of FI on T2-weighted MRI scans. It was originally developed to assess rotator cuff tears [22]. Among the three studies that utilized the Goutallier scale, two reported no correlation between the preoperative paraspinal muscle morphology and postoperative functional outcomes. Interestingly, Pinter et al. [11] reported that patients with higher Goutallier grades experienced greater improvements in the NDI than those with lower-grade degeneration. These findings were explained by the hypothesis that patients with more structurally impaired muscles may benefit more significantly from the enhanced mechanical support provided by the interbody cage used in ACDF.

Sarcopenia has been extensively studied in various medical fields. Cardiac surgery has been shown to significantly increase the risk of both early and late mortality [23]. Similarly, in general surgery, sarcopenia has been identified as a significant predictor of poor postoperative outcomes [24]. In the field of spine surgery, the impact of paraspinal muscle degeneration has been investigated more comprehensively in the lumbar spine than in the cervical spine. Studies have demonstrated that the preoperative degeneration of the lumbar paraspinal muscles is associated with several adverse outcomes, including pedicle screw loosening, proximal junctional kyphosis, adjacent segment disease, and persistent low back pain [25,26,27].

Neck muscles play a crucial role in providing stability to the spinal column, with the deepest layers of both the flexor and extensor muscles being particularly important [19]. The multifidus, semispinalis cervicis, and semispinalis capitis are key extensors that help maintain natural cervical lordosis [28,29]. A prolonged forward head posture can overstretch these muscles, leading to weakness and a diminished ability to counteract forces that promote cervical flexion [30]. This muscular imbalance may contribute to the loss of cervical lordosis and the development of kyphotic deformities, which can in turn lead to neck pain and functional disability [31]. On the anterior side, the deep flexor muscles—particularly the longus colli—complement the action of the extensors by providing anterior spinal stability and counteracting the increased lordotic curvature caused by the weight of the head [32]. Furthermore, the two studies in this review that reported a negative correlation between paraspinal muscle degeneration and functional outcomes identified this relationship specifically for deep flexor or extensor muscle groups [10,13]. These findings suggest that the integrity of these deep stabilizing muscles may be particularly influential for recovery and long-term outcomes following anterior cervical spine surgery.

All six studies included in our review indicated that patients received some form of postoperative physiotherapy. However, none of these studies described a specific rehabilitation regimen or implemented a structured rehabilitation protocol. Rehabilitation as part of conservative treatment plans for CDDD has been studied in relation to its impact on typical symptoms associated with the disease [33]. A randomized controlled trial (RCT) from 2003 demonstrated that, compared with a control group, patients who participated in a targeted exercise program focused on cervical extensor muscles demonstrated greater improvements in the NDI and VAS scores [34]. Conversely, structured postoperative rehabilitation has not consistently been shown to be superior to standard care following cervical spine surgery [35]. Nevertheless, previous studies have indicated that the benefits of strength training may diminish if the training is discontinued after only a few months [36]. Therefore, adherence to an exercise program appears to be particularly important for long-term outcomes. Moreover, another RCT suggested that initiating rehabilitation within 1–2 weeks after surgery leads to greater improvements in the NDI, JOA, and VAS scores for neck pain than usual postoperative care does [37].

Additionally, the studies we reviewed differed considerably in their choice of outcome assessment tools, which may have contributed to the variability in reported results and limited our ability to directly compare findings across studies. Standardized and validated outcome measures are critical for reliably evaluating the effectiveness of surgical interventions. According to the North American Spine Society (NASS) guidelines, recommended outcome measures for assessing the surgical treatment of cervical degenerative disk disease (CDDD) include the Neck Disability Index (NDI), the Visual Analog Scale (VAS) scores for pain, and general health or quality of life measures, such as the SF-12 or SF-36 [38].

### Limitations and Future Studies

This review had several limitations. The number of available studies was small, and the majority of included articles were retrospective in design, with relatively small sample sizes and low levels of evidence. Another major limitation was the high degree of heterogeneity among the studies. The literature varied significantly in terms of patient populations—some studies included patients with myelopathy, and previous research has shown that degenerative cervical myelopathy (DCM) is associated with increased FI in paraspinal muscles at and below the level of spinal cord compression [39], which can affect muscle assessments.

There was inconsistency in the methods used to assess the paraspinal muscles. As prior studies have demonstrated no clear correlation between the muscle cross-sectional area and FI [21], the quantitative evaluation of muscle quality—particularly deep cervical flexors and extensors—may offer the most reliable insight into functional relevance. Future studies should explore how quantitative and qualitative evaluations of paraspinal muscles affect outcomes after cervical spine surgery.

Technical factors during cervical spine surgery, such as the type of surgical visualization used, may affect paraspinal muscle preservation. Exoscope-assisted procedures may offer improved visualization and ergonomics while achieving similar outcomes, which could help reduce iatrogenic muscle trauma [40]. Future studies should evaluate whether the use of exoscopes or other advanced visualization tools contributes to the better preservation of paraspinal musculature.

Finally, the studies included in this review rarely described their postoperative rehabilitation programs in detail. Because exercise programs are an integral part of spine surgery recovery, future studies would be strengthened by incorporating structured and specific training protocols after surgery. Exercise therapy and stabilization programs have been proven to improve muscle mass in patients with neck or back pain [41,42]. Future studies may investigate the role of preoperative rehabilitation in cervical spine surgery—particularly, how it affects the quality of the paraspinal muscles and surgical outcomes.

## 5. Conclusions

We found inconclusive evidence concerning the impact of the condition of the paraspinal muscles on functional outcomes following anterior cervical spine surgery. Two of the six studies revealed that the degeneration of the paraspinal muscles was negatively associated with functional outcomes following anterior cervical spine surgery, whereas one study demonstrated a positive association, and three studies found no association. Well-designed multicenter prospective studies with standardized protocols, consistent criteria for patient selection, reliable assessments of the paraspinal muscles, and structured postoperative care are needed.

## Figures and Tables

**Table 1:** Characteristics of included studies.

Author and Year	Study Design	Follow-Up	Patient Characteristics	Type of Surgery	Muscle Assessment	Outcome Assessment	Level of Evidence	Key Findings
Caffard et al., 2024 [10]	Retrospective cohort	364 days median	104 M, 64 F Mean age 55 years CDDD	ACDF with plate	T2W MRI quantitative assessment	NDI	III	Higher FI In PM muscles correlated with worse improvement in NDI and progression of C2–C7 SVA.
Pinter et al., 2021 [11]	Retrospective cohort	Min. 1 year	34 M, 35 F Mean age 53.1 years CDDD	ACDF	T2W MRI, Goutallier grade at C5/C6 level	NDI, RAND, EQ5D	III	Patients with higher Goutallier grade experienced better improvement in NDI. All groups experienced significant improvement in NDI, RAND, and EQ5D according to the Goutallier grade.
Wang et al., 2022 [12]	Retrospective cohort	Mean 18 months (range 12–75 months)	58 M, 43 F Mean age 50.7 years Single-level CDDD causing symptomatic radiculopathy or myelopathy	ACDF with plate	T2W MRI, Goutallier grade at C5/C6 level	JOA, NDI, VAS	III	On the basis of Goutallier grade, all groups showed significant improvements following surgery in JOA, VAS, and NDI scales. No significant differences were observed between groups in JOA, NDI, and VAS.
Thakar et al., 2019 [13]	Prospective cohort study	Mean 20.02 ± 8.63 months	40 M, 5 F Mean age 40.2 years Single level CDDD radiculomyelopathy or myelopathy	ACD	T2W MRI muscle CSA–VBA, DF to DE ratio	Nurick grade	II	DF area and DF/DE ratio correlated positively with NGI.
He et al., 2023 [14]	Retrospective cohort	Mean 23.4 months (range 12–93 months)	38 M, 72 F Mean age 48.8 years Radiculopathy or/and myelopathy	HS arthroplasty or arthroplasty + ACDF	T2W, Goutallier grade, focused on multifidus belly	JOA, VAS, NDI	III	On the basis of Goutallier grade, all groups showed significant improvements following surgery in JOA, VAS, and NDI. No significant differences were observed between groups in JOA, NDI, and VAS.
Thakar et al., 2014 [15]	Retrospective cohort	Mean 20.48 ± 11.25 months (range 6–90 months)	61 M, 6 F Mean age 52 years Cervical spondylotic myelopathy	Corpectomy with fusion and plating	Muscle CSA–VBA ratio, ratio between DF and DE	Nurick grade	III	No correlation was found between muscle area and functional outcome.

Abbreviations: ACD: anterior cervical discectomy; ACDF: anterior cervical discectomy and fusion; CDDD: cervical degenerative disk disease; CSA: cross-sectional area; DE: deep extensor; DF: deep flexor; EQ5D: EuroQol 5-dimension health questionnaire; FI: fatty infiltration; HS: hybrid surgery (arthroplasty ± ACDF); JOA: Japanese orthopedic association score; MF: multifidus; NDI: neck disability index; NGI: Nurick grade improvement; PM: posteromedial muscles; RAND: RAND 36-item health survey; SVA: sagittal vertical axis; T2W: T2-weighted imaging; VAS: visual analog scale; and VBA: vertebral body area.

## Data Availability

No new data were created or analyzed in this study.

## Supplementary Materials

The following supporting information can be downloaded at: https://www.mdpi.com/article/10.3390/jcm14238453/s1, Table S1: Summary of studies assessed using the Modified Newcastle-Ottawa Scale.; Table S2: Supplemental Material PRISMA Checklist. Reference [43] are cited in the supplementary materials.
