# Supplementary material for: Relationship Between Paraspinal Muscle Degeneration and Functional Outcomes Following Anterior Cervical Spine Surgery for Degenerative Disk Disease: A Systematic Review
Source: J Clin Med. 2025 Nov 28;14(23):8453. doi: 10.3390/jcm14238453 (PMC12693662; doi:10.3390/jcm14238453)
Supplement: Supplementary file 1 [file jcm-14-08453-s001.zip › jcm-3981759-Table S1.pdf]

Table S1. Summary of studies assessed using the Modified Newcastle-Ottawa Scale.

Selection	Exposed Cohort Representative?	Selection of Non-Exposed Cohort	Ascertainment of Exposure	Outcome not Present At study Start?	Study controls for Age (Internal Comparison)	Study Controls for Sex, BMI * (Internal Comparison)	Assessment of Outcome	Timing of Follow-Up (≥12 Months)	Adequate Follow-Up (≥80%) or Not Stated	Score
Caffard et al., 2024 [10]	+	−	+	+	+	+	+	+	−	7/9
Pinter et al., 2021 [11]	+	−	+	+	+	+	+	+	−	7/9
Wang et al., 2022 [12]	+	−	+	+	−	+	+	+	−	6/9
Thakar et al., 2019 [13]	+	−	+	+	+	−	+	+	−	6/9
He et al., 2023 [14]	+	−	+	+	+	+	+	+	−	7/9
Thakar et al., 2014 [15]	+	+	+	+	+	−	+	+	−	7/9
